# Growth Performance, Rumen Fermentation, and Meat Quality of Finishing Lambs Supplemented with Calcium Propionate or Sodium Propionate

**DOI:** 10.3390/vetsci11120604

**Published:** 2024-11-28

**Authors:** Lucero Abigail Velázquez-Cruz, Pedro Abel Hernández-García, Germán David Mendoza-Martínez, Enrique Espinosa-Ayala, Héctor Aarón Lee-Rangel, Gabriela Vázquez-Silva, Pablo Benjamín Razo-Ortíz, Cesar Díaz-Galván, José Felipe Orzuna-Orzuna, María Eugenia de la Torre-Hernández

**Affiliations:** 1 Centro Universitario Amecameca, Universidad Autónoma del Estado de Mexico, Amecameca 56900, Mexico; lucemvz14@gmail.com (L.A.V.-C.); enresayaa1@hotmail.com (E.E.-A.); mvzrazo@gmail.com (P.B.R.-O.); 2 Departamento de Producción Agrícola y Animal, Universidad Autónoma Metropolitana—Xochimilco, Mexico City 04960, Mexico; gmendoza@correo.xoc.uam.mx (G.D.M.-M.); cesarwardi14@gmail.com (C.D.-G.); 3 Facultad de Agronomía y Veterinaria, Centro de Biociencias, Instituto de Investigaciones en Zonas Desérticas, Universidad Autónoma de San Luis Potosí, Soledad de Graciano Sánchez 78000, Mexico; hector.lee@uaslp.mx; 4 Departamento del Hombre y su Ambiente, Universidad Autónoma Metropolitana—Xochimilco, Mexico City 04960, Mexico; gavaz@correo.xoc.uam.mx; 5 Departamento de Zootecnia, Universidad Autónoma Chapingo, Chapingo 56230, Mexico; jforzuna@gmail.com; 6 CONAHCYT-UAM Xochimilco, Universidad Autónoma Metropolitana Xochimilco, Mexico City 04960, Mexico; mdelatorre@correo.xoc.uam.mx

**Keywords:** gluconeogenic precursors, dietary energetics, feed additive, carcass yield

## Abstract

This study aimed to evaluate the effect of dietary supplementation with calcium propionate (CaPr) or sodium propionate (NaPr) on growth performance, ruminal fermentation, and meat quality of finishing lambs. Twenty-seven non-castrated Creole male lambs (24.95 ± 2.15 kg body weight (BW); 4.5 ± 0.5 months old) were randomly assigned to three treatments: (a) CON: basal diet without the addition of CaPr or Na Pr; (b) basal diet + CaPr (10 g/kg DM); and (c) basal diet + NaPr (10 g/kg DM). The data were analyzed using a completely randomized experimental design, with each lamb considered an experimental unit (nine replicates/treatment). Dietary supplementation with CaPr or NaPr did not affect (*p* > 0.05) growth performance or dietary energetics. However, greater (*p* = 0.05) apparent dry matter digestibility was observed in the lambs that consumed the diet with NaPr10. Dietary supplementation with CaPr or NaPr did not affect (*p* > 0.05) ruminal pH or ruminal concentrations of ammonia nitrogen, acetate, propionate, butyrate, and total volatile fatty acids. However, ruminal lactate concentration increased (*p* = 0.01) in lambs consuming the NaPr diet. Hot carcass weight and yield, backfat thickness, meat pH, meat color (L*, a*, and b*), cooking loss, and water holding capacity were not affected by dietary supplementation with CaPr or NaPr. In conclusion, dietary supplementation with 10 g/kg DM of calcium propionate or sodium propionate does not affect growth performance, dietary energetics, ruminal fermentation, and the meat quality of finishing lambs.

## Simple Summary

Including gluconeogenic precursors such as calcium propionate and sodium propionate in ruminant diets has been shown to positively impact animal growth. However, information on the effects of calcium or sodium propionate on dietary energetics, ruminal fermentation, and meat quality of lambs is scarce. This study aimed to evaluate the effects of dietary supplementation with calcium propionate and sodium propionate on growth performance, dietary energetics, ruminal fermentation, and meat quality of finishing lambs. Calcium propionate and sodium propionate do not affect growth performance, dietary energy utilization efficiency, ruminal fermentation, or meat quality of finishing lambs.

## 1. Introduction

Currently, in several countries, the demand for sheep meat has increased [1], which has caused greater use of intensive lamb finishing systems [2]. In these systems, animals are fed diets that contain a high proportion of cereal grains [3], which are essential to satisfy the high energy requirements of lambs during finishing [4]. However, the price of cereal grains is high [5], and when they represent more than 60% of the diet, they can increase the incidence of ruminal acidosis [6], which leads to less feed consumption and weight gain [1]. Therefore, several glucogenic precursors, such as glycerol [7], propylene glycol [8], calcium propionate (CaPr) [9], and sodium propionate (NaPr) [10], have been evaluated as possible alternatives to partially replace cereal grains commonly included in diets for finishing lambs.

According to Liu et al. [11] glucogenic precursors can replace a part of the cereal grains in the diet since they promote the formation of ruminal propionate without negatively affecting the ruminal concentration of acetic and butyric acid. Particularly in finishing lambs, some studies [9,12] show that CaPr and NaPr can be used to replace part of the dietary energy provided by cereal grains. CaPr and NaPr are the propionate salts most studied in ruminants [4,13]. According to Zhang et al. [14], CaPr is a salt produced through a chemical reaction combining propionic acid and calcium hydroxide. After being consumed by ruminants, CaPr dissociates into calcium ions and propionic acid in the rumen [4]. In contrast, NaPr is a salt formed by propionic acid and sodium hydroxide, which can be included in livestock diets to reduce microbial growth [15].

Previous studies [9,12,16] used low to moderate doses (10 and 20 g/kg DM) of CaPr in diets for growing-finishing lambs without decreasing productive performance or feed intake. Similarly, Berthelot et al. [17] and Beiranvand et al. [13] reported that high doses (50 g/kg DM) of NaPr can be added to diets in lambs and calves without adverse effects on growth performance. Mendoza-Martínez et al. [9] estimated that the metabolizable energy (ME) of CaPr is 3.76 Mcal/kg, while NaPr has 2.3 Mcal/kg of ME [10]. The metabolism of CaPr and NaPr is similar; however, information on the effects of NaPr on ruminal fermentation and carcass traits of finishing lambs is scarce. On the other hand, several studies [1,3,12] have evaluated the effects of CaPr on the productive performance and ruminal fermentation of lambs. However, a recent meta-analysis [4] concluded that more studies are needed on the effects of low doses (<20 g/kg DM) of CaPr in finishing lambs fed diets high in grains to obtain reliable conclusions about the treatment effect. Based on the above background, we hypothesize that dietary supplementation with low doses (10 g/kg DM) of CaPr or NaPr will benefit the growth performance, ruminal fermentation, and meat quality of finishing lambs. Therefore, the current study aimed to evaluate the effect of dietary supplementation with calcium propionate (CaPr) and sodium propionate (NaPr) on the growth performance, ruminal fermentation, and meat quality of finishing lambs.

## 2. Materials and Methods

The experimental phase was carried out on the Centro Universitario Amecameca of the Universidad Autónoma del Estado de Mexico (latitude: 19°17′02″ N; longitude: 99°40′41″ W; altitude: 2420 masl). All lamb care procedures were approved by the Research Ethics and Bioethics Committee of the Universidad Autónoma del Estado de Mexico (Protocol #16-05 2023).

### 2.1. Animals and Diets

Twenty-seven non-castrated Creole lambs (24.95 ± 2.15 kg BW; 4.5 ± 0.5 months old) were randomly distributed in three treatments (nine replicates/treatment): (a) CON: diet without addition of CaPr or NaPr; (b) CaPr10: diet added with CaPr (10 g/kg DM); and (c) NaPr10: diet added with NaPr (10 g/kg DM). The CaPr dose (10 g/kg DM) was chosen based on a recent dose–response study [3], in which this dose showed the best growth performance results in finishing lambs. In contrast, the NaPr dose chosen (10 g/kg DM) was equal to the CaPr dose because the composition (propionic acid and calcium content) of both additives is very similar (Table 1 footer) and because no previous dose–response study has identified the most suitable NaPr doses for lambs. Before the beginning of the experimental phase, all lambs were adapted to individual pens and the basal diet for ten days and were subsequently subjected to an experimental phase of 42 days. The individual pens were fully covered by galvanized sheets at a height of 3 m, had a concrete floor, and a total area of 1.8 m^2^ each. All lambs were dewormed orally with 4 mL/lamb of a mixture of Closantel and Albendazole (Closalben-F^®^, Lapisa, Labs, Michoacán, Mexico). Likewise, each lamb was injected intramuscularly with 0.5 mL of vitamins A, D, and E (Vigantol^®^, Bayer, Labs, Mexico City, Mexico). The CaPr and NaPr products used in the current study were purchased from Alimentaria Mexicana Bekarem (Bekarem^®^ S.A. de C.V., Mexico City, Mexico). The experimental diets were formulated based on the NRC requirements [18] to obtain daily weight gains of 300 g. The doses (10 g/kg DM) of CaPr or NaPr were mixed with the diet’s minor ingredients (mineral premix, calcium carbonate, and common salt), and then the combination of these ingredients was mixed with the rest of the ingredients. These diets were provided to the lambs twice daily at 9:30 a.m. and 4:30 p.m. To ensure ad libitum food consumption, a 5% larger amount of food than the previous day was offered daily, while water was offered ad libitum throughout the experimental phase. Table 1 shows the nutritional composition of the diets used, which were isoenergetic and isoproteic. The forage/concentrate ratio of the experimental diets was 82.4/17.6.

Samples of the experimental diets were collected weekly and stored in polyethylene bags at −20 °C. Before chemical analyses, subsamples were thawed for 12 h at room temperature. Subsequently, equal amounts of the subsamples collected weekly were mixed to obtain a composite sample. Samples were dried at 55 °C for 72 h in a forced-air oven and then ground with a Wiley mill (model 4, Arthur Thomas Co. Philadelphia, PA, USA) using a 1 mm sieve. Finally, the contents of dry matter (method 967.03), crude protein (method 981.10), ether extract (method 920.29), and ash (method 942.05) were determined following the procedures described by AOAC [19]. The contents of neutral detergent fiber and acid detergent fiber were determined using the methods of Van Soest et al. [20].

### 2.2. Growth Performance, Dietary Energetics, and Apparent Dry Matter Digestibility

The offered and rejected feed were weighed daily to estimate dry matter intake (DMI). At the beginning (day 0) and at the end (day 42) of the experiment, the lambs were weighed individually, and the body weight (BW) of each was recorded. Before the initial and final weighing, all lambs were fasted for 12 h. The following equation, body weight (BW) × 0.96, proposed by Cannas et al. [21] was used to convert BW to shrunk BW (SBW) and thus adjust BW for gastrointestinal filling. Average daily gain (ADG) was estimated as (final SBW − initial SBW)/42, while feed conversion ratio (FCR) was estimated with DMI/ADG.

Procedures described in previous studies [22,23] were used to estimate observed dietary net energy (NE) for maintenance (ObsNEm), observed dietary NE for weight gain (ObsNEg), the ratio of observed to expected dietary NE for maintenance (OExNEm), and the ratio of observed to expected dietary NE for weight gain (OExNEg). These parameters are used to assess the efficiency of dietary energy utilization in lambs [22,23]. Prior to estimating the values of ObsNEm and ObsNEg, the energy required for maintenance (ME, Mcal/d) and the energy required for gain (EG, Mcal/d) were calculated with the following equations [18]: ME = 0.056 × SBW^0.75^ and EG = ADG × 0.276 × SBW^0.75^. The coefficient of 0.276 was taken from NRC [24], assuming a medium mature weight for male Creole lambs [25]. Subsequently, with the estimated values of ME and EG and with the DMI values observed in the current study, the ObsNEm was calculated using the quadratic formula x = (−b − √(b^2^ − 4ac))/2c, in which x = ObsNEm (Mcal/kg), a = −0.41 EM, b = 0.877 EM + 0.41 DMI + EG, and c = −0.877 DMI [26]. Finally, with the obtained ObsNEm values, ObsNEg was estimated using the following equation [26]: ObsNEg = 0.877 ObsNEm − 0.41.

From day 26 to day 30 of the current study, feces samples were taken daily from each lamb directly from the rectum to estimate apparent DM digestibility. For this, acid-insoluble ashes were used as an internal marker, and the procedures described by Van Keulen and Young [27] were followed.

### 2.3. In Vivo Ruminal Fermentation

At the end of the experimental phase, under pre-prandial conditions, samples of ruminal fluid (50 mL) were taken from each lamb using an esophageal probe. The liquid was collected in an Erlenmeyer flask, filtered with a double layer of gauze, and the ruminal pH was immediately measured using a portable pH meter (model Orion Star A215, Thermo Fisher Scientific^TM^, Waltham, MA, USA). A 3 mL subsample was taken from each lamb’s sample, transferred to a test tube, and 1 mL of 25% metaphosphoric acid was added. The concentration of acetate, propionate, butyrate, and total volatile fatty acids (VFA) was determined using gas chromatography [28] with a Perkin Elmer Clarus 580 equipment with a 30 m × 0.25 mm × 25 µm capillary column (model HP-FFAP, Agilent Technologies, Mexico City, Mexico). On the other hand, the rumen concentration of ammonia nitrogen (NH_3_-N) was determined with the procedures of McCullough [29] using a spectrophotometer (model Genesys 10S UV-VIS, Thermo Fisher ScientificTM, Waltham, MA, USA) at 630 nm. In the current study, the colorimetric method described by Madrid et al. [30] was used to measure lactate concentration in ruminal fluid with the same spectrophotometer used to measure NH_3_-N but at 570 nm.

### 2.4. Carcass Traits and Meat Quality

At the end of the experimental phase and after the last weighing (day 42), five lambs were randomly selected from each treatment, which remained fasted for 12 h and then were sacrificed. The Official Mexican Standard (NOM-033-SAG/ZOO-2014) were used during the lamb slaughter process. Immediately after slaughter, the skin, legs, head, and viscera were removed from the carcass, and the hot carcass weight (HCW) was recorded. The equation (HCW/final BW) × 100 was used to estimate the hot carcass yield (HCY), as reported by Dorantes-Iturbide et al. [22]. To measure the backfat thickness (BFT) of the carcass, a 4 cm perpendicular incision was first made between the 12th and 13th ribs, and then a vernier was used to measure the BFT, as described by Zheng et al. [31].

Immediately after slaughter, meat pH was measured in triplicate between lumbar vertebrae 7 and 10 using a portable pH meter (model HI 99163, Hanna Instruments, Woonsocket, RI, USA) equipped with a puncture electrode [32]. Twenty-four hours after slaughter, the Longissimus dorsi muscle between the 11th and 13th ribs was removed from the carcass of each lamb. These samples were immediately analyzed for color, water-holding capacity (WHC), and cooking loss (CL). A spectrophotometer (model CR-400, Minolta Holdings Inc., Osaka, Japan) was used to measure lightness (L*), redness (a*), and yellowness (b*) in triplicate. Furthermore, the obtained values of a* and b* were used to estimate the Chroma and Hue indices with the equations Chroma = (a*2 + b*2)0.5 and Hue = tan − 1 (b*/a*) × 57.29 [33]. WHC was estimated in triplicate using the method described by Tsai and Ockerman [34]. Briefly, subsamples of 0.5 g of *Longissimus dorsi* muscle were weighed in triplicate, and each was placed between two filter papers. Subsequently, the samples were pressed individually between two 15 × 15 cm^2^ glass plates using a 10 kg weight for 20 min. WHC (%) was calculated with the equation [initial weight–final weight)/initial weight] × 100 [1,35]. The CL was determined in triplicate with the method described by Cañeque et al. [36]. Briefly, 20 g filets were placed in triplicate in sealed polyethylene bags. Subsequently, the samples were heated in a water bath at 75 °C until they reached an internal temperature of 70 °C. Finally, the samples were cooled for 15 min using water at room temperature (approximately 20 °C), they were removed from the bags, excess surface moisture was removed, and they were weighed individually. CL (%) was calculated with the equation [initial weight–final weight)/initial weight] × 100 [1,35].

### 2.5. In Vitro Gas Production

Samples of the experimental diets were taken, dried at 65 °C for 24 h, and ground with a mill using a 1 mm sieve. Of each experimental diet, 500 mg were incubated using 90 mL of culture medium (CM). No additional calcium propionate or sodium propionate was added during in vitro fermentation. The CM was prepared following the description of Cobos and Yokoyama [37]. Briefly, CM contained the following: (1) mineral solution A (6 g of potassium hydrogen phosphate in 1 L of distilled water); (2) mineral solution B (mixture of 6 g of ammonium sulfate, 6 g of potassium hydrogen phosphate, 12 g of sodium chloride, 2.45 g of magnesium sulfate, and 1.6 g of calcium chloride in 1 L of distilled water); (3) reduced cysteine solution (mixture of 2.5 g of L-cysteine in 15 mL of sodium hydroxide solution (2 N), 2.5 g of sodium sulfide, and 0.1 mL of rezarsurin (1%)); and (4) buffer solution of 18% sodium carbonate.

At the end of the in vivo experiment, an esophageal probe was used to take rumen fluid samples (50 mL) from each lamb (under pre-prandial conditions) using a Tygon tube connected to a vacuum pump and a 1000 mL Erlenmeyer flask. These rumen fluid samples were filtered with gauze and immediately transported to the laboratory at 39 °C. Before the start of the in vitro fermentation, individual samples within each treatment were pooled for subsequent use. Subsequently, 500 mg of diet, 10 mL of ruminal fluid, and 90 mL of CM were added to amber flasks. Subsequently, the flasks were hermetically sealed and placed in a water bath at 39 °C. Ten repetitions and three blanks were incubated for each experimental diet for correction. With a manual manometer (scale 0 to 1 kg/cm^2^), the gas pressure was measured at 0, 3, 6, 9, 12, 24, 48, 60, and 72 h of incubation. These data were transformed into gas volume with the following equation [38], V = [P + 0.0495]/0.0185, in which V = gas volume in mL/g MS and P = gas pressure in kg/cm^2^. Gas production kinetics parameters such as maximum gas volume produced (Vmax, mL/g DM), gas production rate (S, mL/h), and lag phase (L, h) were obtained using the volume values of gas accumulated using the following logistic model [39]: Vo = Vm/1 + [exp]^(2 − 4S(t − L)). After 72 h of in vitro incubation, the in vitro dry matter digestibility (IVDMD) of the experimental diets was measured using the residue of the flasks, which was filtered using Whatman No. 41 filter paper and dried at 65 °C for 48 h as reported by Tirado-Estrada et al. [38].

### 2.6. Statistical Analysis

Data were analyzed using a completely randomized experimental design using the PROC GLM procedure of the SAS statistical software [40]. The initial BW was initially tested as a covariate in the growth performance data, while the final BW was tested as a covariate in the carcass traits and meat quality data. However, the initial and final BW values were excluded from the final statistical models because they were not significant (*p* > 0.05). A probability ≤ 0.05 was considered statistically significant, and treatment means were separated using the Tukey test. The final statistical model used was

Y_ijk_ = µ + T_i_ + e_ij_

where Y_ij_ represents the observations, µ = general mean, T_i_ = fixed effect of the ith treatment, and e_ij_ = random error.

## 3. Results

Table 2 shows that dietary supplementation with CaPr or NaPr did not affect (*p* > 0.05) final body weight, DMI, ADG, or FCR. Likewise, dietary supplementation with CaPr or NaPr did not affect (*p* > 0.05) ObsNEm, ObsNEg, OExNEm, and OExNEg.

Table 3 shows that dietary supplementation with CaPr or NaPr did not affect (*p* > 0.05) ruminal pH or ruminal concentrations of acetate, propionate, butyrate, total VFA, and NH_3_-N. However, ruminal lactate concentration increased (*p* = 0.02) in response to dietary NaPr supplementation.

Table 4 shows that dietary supplementation with CaPr or NaPr did not affect (*p* > 0.05) HCW, HCY, or BFT. Likewise, meat pH, L*, a*, b*, Chroma, Hue, WHC, and CL were not affected (*p* > 0.05) in response to dietary supplementation with CaPr or NaPr.

In vitro fermentation parameters are shown in Table 5. Vmax was similar (*p* > 0.05) among diets of all treatments. However, S, L, and IVDMD were lower (*p* < 0.05) in diets supplemented with CaPr and NaPr than in the control diet.

## 4. Discussion

Previous studies [41,42,43] have reported that intraruminal or intravenous infusion of CaPr or NaPr decreases DMI in ruminants. However, in the current study, dietary supplementation with CaPr or NaPr did not significantly affect DMI. This effect was expected since a recent meta-analysis [4] showed that, in lambs, CaPr only decreases DMI when high doses (>20 g/kg DM) are used. Likewise, some studies [10,13,15] have shown that it is possible to include high doses (up to 56 g/kg DM) of NaPr in diets for sheep and cattle without affecting DMI. On the other hand, final BW, ADG, and FCR were not affected in response to the consumption of the CaPr10 or NaPr10 diets. Similarly, previous studies [9,12,44] also did not detect significant changes in final BW, ADG, or FCR of lambs supplemented with various levels (between 10 and 40 g/kg DM for 42 d) of CaPr in the diet. Likewise, other studies reported that the dietary inclusion of high doses (50 and 56 g/kg DM) of NaPr does not affect final BW, ADG, or FCR in lambs [10] and calves [13].

Lambs fed the NaPr10 diet had higher apparent DM digestibility, suggesting that NaPr increases the amount of nutrients absorbed in the digestive tract of finishing lambs [11]. Although this increase in digestibility was not reflected in improved growth performance in the current study, higher nutrient absorption could benefit feed efficiency and lambs’ overall health status, as reported by other authors [13,22].

In the present study, consumption of the CaPr10 and NaPr10 diets did not affect OExNEm or OExNEg. These effects could be related to the similarity detected in the ruminal concentration of total VFA, since these VFA are the primary source of metabolic energy in ruminants [45]. In growing-finishing lambs, the effects of dietary supplementation with CaPr and NaPr on energy metabolism have not been previously evaluated. However, some authors [22,23] indicate that OExNEm and OExNEg values lower and higher than 1.0 indicate low and high efficiency of dietary energy utilization, respectively.

Dietary supplementation with CaPr and NaPr did not significantly affect ruminal pH or ruminal NH_3_-N concentration. These effects were expected since, in lambs, a recent meta-analysis [4] showed that CaPr does not affect ruminal NH_3_-N concentration or ruminal pH regardless of the CaPr dose used. Likewise, it has been reported that supplementation with high doses (50 g/kg DM) of NaPr does not affect rumen pH in calves [13]. The variation in rumen pH serves as an indicator of the internal homeostasis of the rumen [4], while the concentration of NH_3_-N in ruminal fluid depends on the balance between the degradation of the ingested protein and its subsequent use by ruminal microorganisms [45]. In contrast, the NaPr10 diet increased ruminal lactate concentration. The mechanism of action that explains this effect needs to be clarified. However, dietary supplementation with CaPr in dairy cows decreases the ruminal relative abundance of lactate-consuming microorganisms (*Selenomonas ruminantium*) [45]. Therefore, similar effects of NaPr on the ruminal microbiota of finishing lambs could explain the higher ruminal lactate concentration observed. In ruminants, high ruminal lactate concentrations can increase the incidence of ruminal lactic acidosis, a metabolic disorder that causes abdominal distension, loss of appetite, and diarrhea and negatively affects growth performance [4,9].

Dietary supplementation with CaPr and NaPr did not significantly affect the ruminal concentration of acetate, propionate, butyrate, and total VFA. Similarly, previous studies [9,12] also did not detect significant changes in the rumen concentration of acetate, propionate, butyrate, and total VFA of finishing lambs supplemented with CaPr (10 and 20 g/kg DM for 42 d). Similarly, Beiranvand et al. [13] observed that dietary supplementation with high doses (50 g/kg DM for 35 days) of NaPr did not affect the concentration of acetate, propionate, butyrate, and total VFA in the ruminal fluid of growing calves. In contrast, some authors have observed that high doses (≥30 g/kg DM) of CaPr positively modify the ruminal concentration of propionate [46], total VFA, and acetate [44]. Through a meta-analysis, Orzuna-Orzuna and Lara-Bueno [4] demonstrated that CaPr does not affect the rumen concentration of butyrate in lambs regardless of the dose and type of diet used.

The current study showed that dietary supplementation with CaPr or NaPr did not significantly modify HCW, HCY, or BFT. Recent studies [3,46] also did not detect significant changes in HCW, HCY, or BFT of finishing lambs supplemented with increasing doses (10, 20, or 30 g/kg DM per 42 d) of CaPr. Similarly, Berthelot et al. [10] reported that dietary supplementation with NaPr (56 g/kg DM for 40 days) did not affect HCW in finishing lambs. The pH, color, WHC, and CL of small ruminant meat are important parameters that determine its quality [47]. In the present study, no significant changes in pH, color (L*, a*, b*, Chroma, and Hue), WHC, or CL were detected in response to the consumption of the CaPr10 or NaPr10 diets, suggesting that CaPr or NaPr does not affect the quality of sheep meat. To date, the effects of dietary NaPr supplementation on ruminant meat quality have not been evaluated. However, a recent study [1] also did not detect changes in pH, color, WHC, or CL of meat from finishing lambs supplemented with CaPr (10 g/ d) for different periods (14, 28 or 42 d). Likewise, Carrillo-Muro et al. [3] observed that supplementation with low and moderate doses (10 and 20 g/d for 42 d) of CaPr does not affect the pH, L*, a*, b*, WHC, and CL of finishing lamb meat.

Although in the current study dietary supplementation with CaPr or NaPr did not modify meat quality, it is not possible to suggest possible dietary modifications because the effects of CaPr or NaPr on ruminant meat quality have not been evaluated with different types of diet [4]. On the other hand, the use of other doses of CaPr and NaPr could not be suggested either because doses lower than 10 g/kg DM do not improve growth performance in lambs [4], which would not justify their use even if they had a positive impact on meat quality. Likewise, high doses of CaPr improve some meat quality parameters [3] but decrease lamb growth performance [4].

The current study showed that the inclusion of 10 g/kg DM in the diets decreased S, L, and IVDMD values. In contrast, previous studies [48,49,50] did not detect significant changes in S, L, and IVDMD of low- or high-grain diets added with 10 g/kg DM of CaPr. There is no information on the effects of NaPr on the kinetics of in vitro fermentation in lamb diets. However, Ferraro et al. [51] detected that other glucogenic precursors, such as propylene glycol and glycerol, also decrease the in vitro S of some forages (corn and alfalfa) commonly used in sheep feeding. Dietary supplementation with gluconeogenic precursors (e.g., CaPr and NaPr) decreases the relative abundance of soluble and structural carbohydrate-degrading ruminal microorganisms [45], which would explain the lower S values observed in the current study for the CaPr10 and NaPr10 diets. On the other hand, the lower L values in the CaPr10 and NaPr10 diets observed in the current study could be explained by the rapid rate with which ruminal microorganisms can ferment CaPr and NaPr [49,50]. Furthermore, the lower IVDMD at 72 h of incubation observed in the CaPr10 and NaPr10 diets could be explained by the observed reduction in S values since, according to Castañeda-Trujano et al. [52], there is a positive correlation (r = 0.63) between the in vitro values of S and IVDMD at 72 h of incubation.

## 5. Conclusions

Dietary supplementation with low doses (10 g/kg DM) of calcium propionate or sodium propionate does not improve growth performance, ruminal fermentation, or meat quality of finishing lambs. Therefore, dietary supplementation with low doses (10 g/kg DM) of calcium propionate or sodium propionate is not recommended in finishing lambs. In vitro results indicate that adding calcium propionate or sodium propionate in diets for finishing lambs affects fermentation kinetics by reducing the gas production rate.

## Figures and Tables

**Table 1:** Composition of the experimental diets.

	CON	Calcium Propionate	Sodium Propionate
Ingredients (g/kg of diet)			
Ground corn	159	149	149
Ground sorghum	360	360	360
Corn stover	176	176	176
Soybean meal	220	220	220
Cane molasses	60	60	60
Urea	10	10	10
Buffer	10	10	10
Calcium propionate ^1^	0	10	0
Sodium propionate ^2^	0	0	10
Mineral premix ^3^	5	5	5
Chemical composition (g/kg dry matter)			
Dry matter	889.2	888.9	889.1
Organic matter	829.8	828.4	829.3
Crude protein	158.3	157.7	157.9
Neutral detergent fiber	179.6	179.9	180.2
Acid detergent fiber	103.5	102.8	102.1
Ether extract	24.9	25.2	24.7
Ash	58.9	58.5	59.3
Calculated net energy, Mcal/kg			
Maintenance ^4^	1.77	1.77	1.77
Gain ^4^	1.24	1.24	1.24

CON: diet without addition of calcium propionate (CaPr) or sodium propionate (NaPr); calcium propionate: diet added with CaPr (10 g/kg DM); sodium propionate: diet added with NaPr (10 g/kg DM); DM, dry matter. ^1^ Propionic acid 780 g/kg, Ca 180 g/kg. ^2^ Propionic acid 800 g/kg, Na 200 g/kg. ^3^ Mineral premix: 27% calcium, 6.5% sodium, 4.2% sulfur, 3% phosphorus, 0.75% magnesium, 0.05% potassium, 978 mg/kg iron, 160 ppm iodine, 15 ppm cobalt, 25 ppm zinc, 5 ppm copper, 2.25 ppm molybdenum, 1 ppm selenium. ^4^ The calculation is based on NRC [18].

**Table 2:** Growth performance and dietary energetics of finishing lambs supplemented with CaPr or NaPr.

	Treatments				
	CON	CaPr10	NaPr10	SEM	*p* Value
*Growth performance*					
Initial body weight, kg	24.82	25.06	25.25	1.73	0.98
Final body weight (BW), kg	35.66	35.46	35.06	2.00	0.97
Dry matter intake (DMI), kg/d	1.47	1.36	1.47	0.07	0.50
Average daily gain (ADG), kg/d	0.25	0.24	0.23	0.02	0.72
Feed conversion ratio (FCR), kg/kg	5.87	5.76	6.66	0.48	0.36
Apparent DM digestibility, g/100 g	80.18 ^b^	80.80 ^b^	83.77 ^a^	1.07	0.05
*Observed dietary net energy, Mcal/kg of DM*					
Maintenance (ObsNEm)	1.64	1.72	1.55	0.06	0.41
Gain (ObsNEg)	1.02	1.09	0.95	0.06	0.41
*Observed to expected diet net energy, Mcal/kg of DM*					
Maintenance (OExNEm)	0.99	1.06	0.95	0.04	0.41
Gain (OExNEg)	1.04	1.18	0.93	0.10	0.41

CON: diet without addition of calcium propionate (CaPr) or sodium propionate (NaPr); CaPr10: diet added with CaPr (10 g/kg DM); NaPr10: diet added with NaPr (10 g/kg DM); ^a, b^: when superscripts differ within a row, it indicates significant differences (*p* ≤ 0.05) between treatments; SEM: standard error of mean.

**Table 3:** Ruminal fermentation of finishing lambs supplemented with CaPr or NaPr.

	Treatments				
	CON	CaPr10	NaPr10	SEM	*p* Value
Ruminal pH	6.99	6.99	7.00	0.06	0.99
Ammonia nitrogen (NH_3_-N), mg/dL	6.68	6.69	7.61	0.80	0.59
Ruminal lactate, mg/mL	0.61 ^b^	0.99 ^ab^	1.59 ^a^	0.19	0.02
Total volatile fatty acids (VFA), mmol/L	37.99	39.69	33.88	3.99	0.59
Acetate, mol/100 mol	62.42	65.10	65.27	1.40	0.29
Propionate, mol/100 mol	28.59	23.82	23.91	2.17	0.23
Butyrate, mol/100 mol	8.97	11.07	10.81	1.12	0.37

CON: diet without addition of calcium propionate (CaPr) or sodium propionate (NaPr); CaPr10: diet added with CaPr (10 g/kg DM); NaPr10: diet added with NaPr (10 g/kg DM); ^a, b^: when superscripts differ within a row, it indicates significant differences (*p* ≤ 0.05) between treatments; SEM: standard error of mean.

**Table 4:** Carcass and meat characteristics of finishing lambs supplemented with CaPr or NaPr.

	Treatments				
	CON	CaPr10	NaPr10	SEM	*p* Value
Hot carcass weight (HCW), kg	16.74	17.22	16.18	0.67	0.56
Hot carcass yield (HCY), %	48.65	51.02	51.07	0.91	0.14
Backfat thickness (BFT), cm	0.89	0.80	0.91	0.07	0.56
Meat pH	6.51	6.45	6.58	0.06	0.42
Lightness (L*)	24.23	23.89	24.99	1.25	0.82
Redness (a*)	13.19	12.71	11.97	0.51	0.27
Yellowness (b*)	6.44	6.23	6.36	0.31	0.91
Chroma	14.68	14.16	13.16	0.52	0.37
Hue	26.03	26.11	28.06	1.39	0.52
Water-holding capacity (WHC), %	41.00	39.85	39.83	0.60	0.35
Cook loss (CL), %	25.43	24.61	27.77	0.96	0.96

CON: diet without addition of calcium propionate (CaPr) or sodium propionate (NaPr); CaPr10: diet added with CaPr (10 g/kg DM); NaPr10: diet added with NaPr (10 g/kg DM); SEM: standard error of mean.

**Table 5:** In vitro fermentation parameters of diets added with CaPr or NaPr.

	Treatments				
	CON	CaPr10	NaPr10	SEM	*p* Value
Maximum gas volume produced (Vmax), mL/g DM	186.86	186.73	190.86	3.05	0.13
Gas production rate (S), mL/h	0.070 ^a^	0.063 ^b^	0.060 ^c^	0.002	0.02
Lag phase (L), h	1.60 ^a^	1.20 ^b^	0.82 ^c^	0.16	0.05
In vitro dry matter digestibility (IVDMD), g/100 g	83.83 ^a^	80.77 ^b^	78.62 ^c^	0.63	0.003

CON: diet without addition of calcium propionate (CaPr) or sodium propionate (NaPr); CaPr10: diet added with CaPr (10 g/kg DM); NaPr10: diet added with NaPr (10 g/kg DM); ^a, b, c^: when superscripts differ within a row, it indicates significant differences (*p* ≤ 0.05) between treatments; SEM: standard error of mean.

## Data Availability

The data are contained within this article.
